# Autoimmune Inner Ear Disease: Immune Biomarkers, Audiovestibular Aspects, and Therapeutic Modalities of Cogan's Syndrome

**DOI:** 10.1155/2018/1498640

**Published:** 2018-04-23

**Authors:** Oded Shamriz, Yuval Tal, Menachem Gross

**Affiliations:** ^1^Pediatric Division, Hadassah Hebrew-University Medical Center, Jerusalem, Israel; ^2^Internal Medicine Division, Hadassah Hebrew-University Medical Center, Jerusalem, Israel; ^3^Allergy and Clinical Immunology Unit, Hadassah Hebrew-University Medical Center, Jerusalem, Israel; ^4^Department of Otolaryngology-Head and Neck Surgery, Hadassah Hebrew-University Medical Center, Jerusalem, Israel

## Abstract

Cogan's syndrome (CS) is a rare autoimmune disorder characterized by audiovestibular dysfunction and ocular inflammation. Currently, there is no specific serum autoantibody used in the diagnostic workup of CS. Treatment is based on immunosuppressive agents, mainly corticosteroids as first-line choice. Recently, novel therapeutic modalities in CS have emerged. These include tumor necrosis factor-*α* inhibitors and other biologicals. Despite medical treatment, hearing loss may progress to irreversible bilateral profound SNHL in approximately half of CS patients resulting in candidacy for cochlear implantation (CI). Due to the inflammatory nature of the disease that is causing endosteal reaction with partial obliteration or complete neoossification of the intracochlear ducts, early CI is recommended. CI provides excellent and stable hearing rehabilitation with high score of word and sentence recognition. In this review, we will discuss different aspects of CS including clinical presentation, diagnosis, treatment, and future directives.

## 1. Introduction

Classical definition of autoimmune inner ear disease (AIED) has been suggested as a disorder with bilateral sensorineural hearing loss (SNHL) progressing over a period of 3 to 90 days, which showed response to steroid treatment [1]. Suggested mechanisms include humoral, as well as cellular-mediated responses with upregulation and expression of different cytokines, such as interleukin- (IL-) 1*β* and interstitial cell adhesion molecule-1 (ICAM-1) [2]. Many systemic autoimmune diseases may be associated with bilateral rapidly progressive SNHL and vestibular symptoms that clinically resemble AIED.

Within the group of AIED, Cogan's syndrome (CS) is of special interest. Typical CS is characterized by inflammation of the eyes and inner ears, manifesting as interstitial keratitis (IK) and audiovestibulary dysfunction (AVD), respectively [3]. Association with systemic vasculitis is well described [4]. CS is believed to have an autoimmune aetiology, although many questions regarding aetiopathogenesis remain unanswered.

As current understanding of possible causes, disease course, and available biologic treatments is limited, a comprehensive review of the existing literature concerning CS is needed. In this review, we will uncover different clinical audiovestibular aspects, immune mechanisms, and therapeutic modalities and try to shed some light on this rare autoimmune disease.

## 2. Epidemiology of Cogan's Syndrome

CS is a rare disorder with approximately 250 cases reported so far [5]. It affects mainly young Caucasian adults in their third decade of life [6], although cases of CS were reported in children and in the elderly. In one study that analysed data from a cohort of 78 CS patients, median age of disease onset was 25 years and ranged between 5 and 63 years [7]. In large cohorts published, there is no specific gender predominance [8].

## 3. The Clinical Spectrum of Cogan's Syndrome

Mandatory diagnostic criteria of CS consist of SNHL, inflammatory ocular symptoms, and ruling out any other causes of inflammation or infection, such as tuberculosis and syphilis [6].

CS is classified as having a “typical” and an “atypical” presentation. Typical CS, as it was first described in 1945, consists of IK and AVD including Meniere-like episodes and SNHL [9]. In typical CS, inner ear symptoms occur within a time period of 2 years from ocular symptoms [3]. Atypical CS manifests with non-IK inflammatory ocular symptoms. These comprise glaucoma, conjunctivitis, and episcleritis [10]. Uveitis is another ocular manifestation of atypical CS and was reported even in children [11], alerting physicians to be aware of the association between uveitis and SNHL in the context of atypical CS.

Systemic manifestations are more common in atypical CS [3]. Fever, headaches, polyarthralgia and arthritis, myalgia, anorexia, and gastrointestinal (GI) symptoms were previously described in CS patients [12]. Systemic vasculitis is seen in 15–21% of the patients [6]. Aortic root vasculitis, which is reported in 10% of CS patients, can result in life-threatening complications, such as aortic aneurysms, dissection, and insufficiency [13–15]. Mitral insufficiency was also reported [16]. Other organs, such as the kidneys and brain, may be affected by systemic vasculitis in CS [17], and CS patients with stroke have been reported [18].

Interestingly, review of the literature reveals a coexistence between CS and other autoimmune diseases. This includes the presence of atypical CS with granulomatosis with polyangiitis (Wegener's granulomatosis) [19], rheumatoid arthritis [20], and tubulointerstitial nephritis and uveitis (TINU syndrome) [21]. One study reported of 4 inflammatory bowel disease (IBD) patients presenting with CS symptoms, including SNHL and ocular inflammation, following GI symptoms [22]. Another large international multicenter study supported these findings and described 22 CS-IBD patients; 50% of them had GI symptoms before CS onset [23]. This coexistence of CS with other autoimmune diseases constitutes a clue for its autoimmune pathogenesis.

## 4. Autoantibodies and Serological Markers in Cogan's Syndrome

Currently, no specific serological biomarker is available in the routine diagnostic workup of CS. Moreover, the absence of serum autoantibodies does not rule out CS diagnosis [5].

However, several autoantibodies have previously been associated with CS (Table 1). In 2003, researchers from Italy identified autoantibodies produced in CS patients against a “Cogan peptide,” which shared homology with laminin, connexin 26, cell density-enhanced protein tyrosine phosphatase-1 (DEP-1/CD148), SSA/Ro, and reovirus III major core protein lambda 1. Injection of these autoantibodies into mice resulted in vasculitis and ocular symptoms. Furthermore, administering the Cogan peptide into rabbits has resulted in the development of SNHL [24].

As autoimmune aetiology of CS became more likely, a search for other autoantibodies was conducted. Anti-heat shock protein- (HSP-) 70 was suggested to be a strong candidate. Antibodies to both HSP-70 and inner ear 68 kDa antigen were isolated from an experimental autoimmune SNHL model of guinea pigs and patients with progressive SNHL and were suggested to be a marker for AIED [25]. In 2007, another study tested 14 CS patients for anti-HSP-70 serum titers and found that 50% of them were positive, as compared to only 4% in the control group [26]. A cohort of 38 CS patients, 55 autoimmune SNHL patients, and 19 control subjects found that positivity of anti-HSP-70 was highest among typical CS patients (92.9%), followed by ASNHL (52.7%), atypical CS (16.6%), and control (5.2%) groups [27].

Finally, one should also mention general autoimmune markers. These include anti-neutrophil cytoplasmic antibodies (ANCA), anti-nuclear antibodies (ANA), and rheumatoid factor (RF). ANCA are directed against myeloperoxidase (C-ANCA) and proteinase-3 (P-ANCA). Their association with numerous autoimmune diseases, including ulcerative colitis and vasculitides, is well established [28, 29]. Regarding CS, review of the literature yields several case reports of ANCA-associated CS, especially in the context of glomerulonephritis and renal vasculitis [30–33]. ANA and RF were also identified in sporadic CS cases [34]. However, positivity of ANCA, RF, and ANA among CS patients in large studies was found to be low [26], as well as with other general autoimmune markers, such as circulating anticoagulant antibodies, anti-cardiolipin antibodies, and cryoglobulins [35].

It appears that the sensitivity and specificity of serum biomarkers of CS are still not sufficient, and thus the search for a routine laboratory test that will support CS diagnosis is still not completed.

## 5. Biological Therapy in Cogan's Syndrome

Treatment of CS is challenging and particularly concerns AVD. Data regarding immunosuppressive treatment of CS is scarce and mainly relies on case reports and case series [6]. First-line therapy in order to achieve remission in CS remains high-dosage corticosteroids. Treatment failure may necessitate the addition of other immunosuppressive agents, such as methotrexate, cyclophosphamide, azathioprine, and cyclosporine A, which were shown to have a favourable outcome than corticosteroid monotherapy [36, 37]. In a systematic review, the use of methotrexate in CS was most common, as a nonsteroidal immunosuppressive agent [38].

However, there are increasing reports of CS patients treated successfully with different biological drugs (Table 2). Tumor necrosis factor (TNF-*α*) inhibitory agents are increasingly being used in autoimmune diseases, such as IBD. Among that group, data regarding treatment with infliximab, a chimeric anti-TNF-*α* monoclonal antibody, seems to be the most extensive. Review of the literature reveals 12 CS patients treated with infliximab [6, 39–45], of which one patient failed to achieve clinical remission [39]. Data regarding age and gender of patients was available in 11 patients. Five males and 6 females with a mean age of 39.2 (range: 16 to 67) years were reported. AVD was noted in 8 patients, five of which also suffered from IK. Three patients presented with a non-IK ocular manifestation (two patients with scleritis and one with branch retinal arterial occlusion, glaucoma, and anterior uveitis). Infliximab doses were described in 8 patients and included a regimen of 3 mg/kg in most cases. These encouraging reports of infliximab use in CS correspond with a French nationwide study, and systematic review of the literature found that infliximab was more effective achieving AVD remission than corticosteroids or DMARDS alone. Moreover, the use of infliximab was found to predict AVD response [44]. Tayer-Shifman et al. suggested that considering treatment with infliximab after treatment with corticosteroids showed lack of response to corticosteroids within 2-3 weeks, steroid dependency of 10 mg/day, or contraindication for the continuation of corticosteroids. First-line therapy with infliximab was recommended by the authors in cases of severe ocular inflammation, rapid hearing loss, bilateral ear involvement, and systemic manifestations or if contraindicated for starting corticosteroid treatment [6].

There are only few reports of the use of other types of TNF-*α* inhibitors in CS. Etanercept, a TNF-*α* receptor fusion protein, was also shown to improve hearing loss in 2 out of 3 CS patients in a multicenter study [46]. Two other reports described failure to achieve clinical response by using adalimumab, an anti-TNF-*α* monoclonal antibody, in a 69-year-old man [47] and a 25-year-old woman with CS [48].

We found 3 case reports, in which rituximab, a chimeric anti-CD20 antibody, was used in CS. Among them, improvement in hearing loss was noted in only one patient [48]. Lack of efficacy was noted in a patient with atypical CS and non-Hodgkin's lymphoma [45] and in a patient with steroids and azathioprine-refractory CS [49].

Finally, one can find only two case reports describing treatment of adult CS patients with tocilizumab, a humanized anti-IL-6 receptor monoclonal antibody. Both patients presented with SNHL and uveitis [47, 50]. One patient suffered from a systemic disease including aortitis, meningitis, panniculitis, and seronegative arthritis and achieved clinical remission with tocilizumab after failed treatment with high dosage of prednisone, methotrexate, cyclosporine, azathioprine, and adalimumab [50]. Another CS patient was treated with tocilizumab but developed bilateral patchy acute lung injury [47].

## 6. Hearing Prognosis and Otologic Surgical Interventions

CS has a poor prognosis when eye, ear, and cardiovascular complications occur. Most of the untreated patients have moderate, severe, or profound hearing loss at 5-year follow-up [7]. In a literature review of 111 cases by Grasland et al., 54% and 37% of cases with typical and atypical Cogan syndrome remained deaf in both ears despite treatment [35]. Gluth et al. reported profound hearing loss in 52% of CS patients despite immunosuppressive therapy [51].

In patients with bilateral profound SNHL in whom no benefits are obtained from conventional hearing aids, cochlear implantation (CI) is a highly effective hearing rehabilitation modality. However, some clinical reports of patients with CS who have undergone CI describe major surgical issues. CI in CS patients may be technically challenged due to inflammatory endosteal reaction leading to partial obliteration or complete neoossification of the intracochlear ducts [52–55]. Due to this tendency, early cochlear implantation should be recommended in patients with bilateral profound SNHL. Postoperative wound healing problems have been also described in CS patients who have undergone CI [56–58]. Skin atrophy from long-term corticosteroid and immunosuppressant therapy and ischemia caused by vasculitis may be risk factors that contributed to the wound healing complication.

Short-term post-CI hearing outcomes in postlingual CS patients have been described in few clinical reports. Although deterioration of auditory performance after CI has been described by Bovo et al., presumably secondary to apposition or progression of new bone formation in the cochlea, which in turn increases the distance of the CI electrode array from neural structures, most authors agree to the fact that prognosis of cochlear implantation with regard to hearing results is excellent [58–61].

There are only two studies on long-term post-CI hearing outcomes in postlingual CS patients. Kontorinis et al. reported the long-term outcomes of four patients with CS (average follow-up of 9.25 years) providing evidence of hearing outcome's persistence [57].

These four patients achieved mean scores of 78.7 and 92.4% on word and sentence recognition tests, respectively. At their last evaluation, the mean word score was 80%, whereas the mean sentence score was 96.6%. Long-term study on 12 CS patients over 5 years of CI use by Bacciu et al. revealed that CI is safe in the long term and provides excellent and stable hearing results, with group means for word and sentence recognition tests 94 and 96.3%, respectively [62]. The data from these two long-term follow-up studies demonstrated that patients with CS receive significant open-set speech recognition benefits from a CI that remain stable in the long term.

## 7. Future Perspective

Human adipose-derived mesenchymal stem cells (hAdMSC) are known to have immunomodulatory properties. Their use in allograft transplantations was previously reported [63]. Interestingly, several animal models have shown promising results using hAdMSC in various autoimmune diseases. This includes murine models of rheumatoid arthritis [64], experimental autoimmune encephalomyelitis (EAE; animal model of multiple sclerosis) [65], and systemic lupus erythematosus [66]. Reduction of proinflammatory cytokines, such as TNF-*α*, IL-1, and IL-6 [67], and induced production of the anti-inflammatory cytokine IL-10 [66] by hAdMSC constitute a possible immune mechanism.

Several animal models have studied the use of hAdMSC in autoimmune SNHL. Experimental SNHL mice were treated with hAdMSC and demonstrated improvement in hearing parameters, increase in regulatory CD25+ FOXP3+ T cells and IL-10, and decrease in T helper (Th)1/Th17 cellular response [68]. These results were supported by another murine model of autoimmune SNHL, with restoration of hearing loss and similar findings in the modulation of the immune cellular components of Th1/Th17 and regulatory CD25+ FOXP3+ T cells, as well as in induction of IL-10 [69].

Clinical trials regarding hAdMSC use in autoimmune SNHL are scarce. One study examined 10 patients with autoimmune diseases, among them a 19-year-old woman with progressive AIED, in which treatment with hAdMSC demonstrated hearing improvement with a follow-up period of 11 months [70]. However, reviewing the literature, we found no reports of studies regarding hAdMSC use in CS. Applying current data from animal models of autoimmune diseases, including autoimmune SNHL, on CS necessitates further studies that will evaluate efficacy and safety of this novel therapeutic modality.

## 8. Conclusions

CS pathogenesis is still not fully understood; however, an autoimmune underlying mechanism is probably responsible for disease onset. Diagnosis must rely on clinical findings, as laboratory markers are not fully accepted and routinely used. The use of steroids, with or without a combination with another immunosuppressive agent, is needed to achieve initial remission. As biological treatments and hAdMSC therapy develop, their increasing use in autoimmune diseases shows encouraging results, and thus new therapeutic modalities in CS are introduced. Early CI should be recommended in patients with bilateral profound SNHL because of the tendency for partial obliteration or complete neoossification of the cochlea. CI provides excellent and stable hearing rehabilitation with long-term follow-up in most patients.

## Figures and Tables

**Table 1:** Autoantibodies associated with Cogan's syndrome.

Autoantibody	Antigen location	Antigen function	References	
			Human studies: number of CS patients/study design/country of publication	Animal models
Anti-HSP-70	Not specific	Protein folding and ubiquitin-mediated degradation; protection from cell stress	38/case control/Italy [27]	—
			14/case control/Italy [26]	
			8/case control/USA [71]	

ANCA	Neutrophils	Proteolysis (PR3)	5/case reports/France [72]; Japan [32, 73]; USA [30]; the Netherlands [33]	—
		Production of oxidative free radicals (MPO)		

Anti-Cogan peptide	Endothelial cells in the inner ear, lymphocytes, and kidney	Contact inhibition of cell growth; homolog with laminin, connexin 26, SSA/Ro, reovirus III major core protein lambda1, and cell density-enhanced protein tyrosine phosphatase-1 (DEP-1/CD148)	8/case control/Italy [24]	Rabbits and mice [24]

HSP-70: heat shock protein-70; CS: Cogan's syndrome; ANCA: anti-neutrophil cytoplasmic antibody; PR3: proteinase-3; MPO: myeloperoxidase.

**Table 2:** Evidence for treatment of Cogan's syndrome with biological agents.

Biological agent	Immune mechanism	Number of CS patients reported to be treated with biotherapy	Age (years)/gender/clinical presentation	Biologic dosage and regimen	Number of CS patients that responded to biotherapy	Study design	Country of publication	Ref
Infliximab	Chimeric anti-TNF-*α* monoclonal antibody	2	33/M/AVD, IK	300 mg × 1/month	2	CR	Switzerland	[42]
			49/M/SNHL	300 mg × 6/week				
		3	30/F/AVD, IK	3 mg/Kg at weeks 0, 2, 6, 8, and then every 8 weeks	3	CR	Italy	[40]
			29/M/AVD, IK	NA^∗^				
			35/M/SNHL, scleritis	NA				
		2	37/F/AVD, IK	3 mg/kg at 0, 2, and 6 weeks	1	CR	USA	[39]
			36/F/AVD, IK	3 mg/kg for 4 months				
		1	16/M/TINU, SNHL, BRAO, glaucoma, and uveitis	900 mg at 0, 3, and 5 weeks	1	CR	USA	[41]
		1	48/F/AVD	3 mg/kg at 0 and 3 weeks and then every 8 weeks	1	CR	Spain	[43]
		1	51/F/SNHL	3 mg/kg every 8 weeks for 3 years^∗∗^	1	CR	Israel	[6]
		1	NA/NA/AVD, scleritis	NA	1	CR	Switzerland	[74]
		1	67/F/AVD	NA	1	CR	Greece	[45]

Etanercept	TNF-*α* receptor fusion protein	3	NA	25 mg × 2/week for 24 weeks	2	CR	USA	[46]

Adalimumab	Anti-TNF-*α* monoclonal antibody	1	69/M/SNHL, iritis, aortitis, meningitis, panniculitis, and seronegative arthritis	40 mg × 1/week for 2 weeks	0	CR	Japan	[50]
		1	25/F/AVD, conjunctivitis, IK	40 mg × 1/week for 6 months	0	CR	Italy	[48]

Rituximab	Anti-CD20 monoclonal antibody	1	25/F/AVD, conjunctivitis, IK	500 mg × 1/week for 4 weeks	1	CR	Italy	[48]
		1	67/F/AVD	NA	0	CR	Greece	[45]
		1	43/F/AVD, IK	375 mg/m^2^ × 1/week for 4 weeks	0	CR	USA	[49]

Tocilizumab	Humanized anti-IL-6 receptor monoclonal antibody	1	69/M/SNHL, iritis, aortitis, meningitis, panniculitis, and seronegative arthritis	8 mg/kg × 1/month	1	CR	Japan	[50]
		1	59/M/SNHL, anterior uveitis	162 mg × 1/week for 2 weeks	0	CR	USA	[47]

CS: Cogan's syndrome; Ref: references; M: male; F: female; AVD: audiovestibulary dysfunction; SNHL: sensorineural hearing loss; IK: interstitial keratitis; TNF-*α*: tumor necrosis factor-*α*; CD: cluster of differentiation; IL: interleukin; CR: case report; NA: data is not available; TINU: tubulointerstitial nephritis and uveitis syndrome; BRAO: branch retinal artery occlusion. ^∗^Dosage is noted as given according to “international protocol.” ^∗∗^Unpublished data.
